# Costing of a Combination Intervention (Kyaterekera) Addressing Sexual Risk-Taking Behaviors among Vulnerable Women in Southern Uganda

**DOI:** 10.4269/ajtmh.23-0485

**Published:** 2024-04-02

**Authors:** Yesim Tozan, Joshua Kiyingi, Sooyoung Kim, Josephine Nabayinda, Flavia Namuwonge, Edward Nsubuga, Fatuma Nakabuye, Ozge Bahar Sensoy, Proscovia Nabunya, Larissa Jennings Mayo-Wilson, Mary M. McKay, Susan S. Witte, Fred M. Ssewamala

**Affiliations:** ^1^ School of Global Public Health, New York University, New York, New York;; ^2^ Brown School, Washington University in St. Louis, St. Louis, Missouri;; ^3^ International Center for Child Health and Development, Masaka, Uganda;; ^4^ Gillings School of Global Public Health, University of North Carolina at Chapel Hill, Chapel Hill, North Carolina;; ^5^ Columbia University School of Social Work, New York, New York

## Abstract

In Uganda, women engaged in sex work (WESW) are a marginalized population at the intersection of multiple vulnerabilities. The Kyaterekera intervention is targeted at WESW in Rakai and the greater Masaka regions in Uganda and combines a traditional HIV risk-reduction approach with a savings-led economic empowerment intervention and financial literacy training. We estimated the economic costs of the Kyaterekera intervention from a program provider perspective using a prospective activity-based micro-costing method. All program activities and resource uses were measured and valued across the control arm receiving a traditional HIV risk-reduction intervention and the treatment arm receiving a matched individual development savings account and financial literacy training on top of HIV risk reduction. The total per-participant cost by arm was adjusted for inflation and discounted at an annual rate of 3% and presented in 2019 US dollars. The total per-participant costs of the control and intervention arms were estimated at $323 and $1,435, respectively, using the per-protocol sample. When calculated based on the intent-to-treat sample, the per-participant costs were reduced to $183 and $588, respectively. The key cost drivers were the capital invested in individual development accounts and personnel and transportation costs for program operations, linked to WESW’s higher mobility and the dispersed pattern of hot spot locations. The findings provide evidence of the economic costs of implementing a targeted intervention for this marginalized population in resource-constrained settings and shed light on the scale of potential investment needed to better achieve the health equity goal of HIV prevention strategies.

## INTRODUCTION

Uganda has a high HIV burden, with an estimated 1.4 million people currently living with the disease in the country.^1^ Despite the significant progress made in reducing HIV transmission and incidence over the past decade, key populations such as women engaged in sex work (WESW) have a particularly high HIV risk^2^^–^^4^ and bear a disproportionate burden of the disease across the world.^1^^,^^5^ In Uganda, this reality is manifested by a markedly high HIV prevalence of 31.3% among WESW compared with an overall prevalence rate of 5.2% in the general adult (15–49 years) population.^1^ Women engaged in sex work also account for 18% of all new HIV infections in the country.^5^ This key population is at the intersection of a myriad of vulnerabilities, including sex inequality, sex-based violence, financial instability, stigma, and a lack of appropriate access to healthcare and means of HIV prevention.^2^^–^^4^

Traditionally, HIV risk-reduction approaches have focused on community sensitization and promotion of safe sex practices through behavioral change. However, mounting evidence has shown that this traditional approach alone is insufficient to effectively reduce HIV risk among high-risk populations such as WESW.^6^^–^^8^ Some of the major barriers to effectiveness include financial hardship and a lack of economic autonomy.^9^^–^^11^ For example, in Uganda and other sub-Saharan African countries, poverty is the most commonly stated reason for commercial sex work, and transactional sex is a survival strategy among women to meet their basic needs.^2^^,^^12^^,^^13^ Sustained financial hardship over time not only motivates women to continue to engage in transactional sex and commercial sex work, but it also limits their negotiating power, particularly among WESW, further increasing their exposure to risky sexual behaviors and HIV infection.^14^

Against this background, a growing body of literature suggests that HIV prevention interventions targeting this key population should address risk factors beyond the individual level to prove effective.^15^^,^^16^ Combining savings-led economic empowerment interventions with traditional HIV prevention and treatment approaches has been effective and cost-effective in low-resource settings. In Uganda, this approach has proven successful in improving knowledge of sexual risk behaviors and reducing sexual risk behavior intentions among adolescents and young women.^17^^,^^18^ In Mongolia, it has demonstrated efficacy in reducing sexual risk behaviors among WESW.^16^^,^^19^^–^^21^ In addition, these combination approaches have been shown to be effective in improving antiretroviral therapy (ART) adherence and mental health outcomes among HIV-infected adolescents.^17^^,^^22^^–^^28^ In light of this evidence base, a 5-year NIH-funded longitudinal study was conducted to assess the efficacy of a combination intervention, Kyaterekera, adding savings, financial literacy, and vocational training/mentorship to a traditional HIV risk-reduction intervention in reducing new incidences of HIV infections and sexually transmitted infections (STIs) among WESW in Rakai and the greater Masaka regions in Uganda.^29^ Savings-led approaches for economic empowerment align with Uganda’s Government Vision 2040, which calls for strategic investments to achieve financial inclusion of poor vulnerable groups and are hence an area of focus for rigorous evaluations.^30^ Alongside an assessment of intervention effectiveness, intervention costing is crucial for resource allocation decisions and priority setting, particularly in low-resource settings in the face of competing public health demands. This study contributes to currently limited economic data in this area by estimating the costs of the Kyaterekera intervention and supports the use of evidence-based economic empowerment interventions in low-resource settings.

## MATERIALS AND METHODS

### Study population, setting, and design.

The Kyaterekera study was designed as a randomized controlled trial that originally consisted of three study arms: a control arm with a traditional HIV risk-reduction intervention; a first intervention arm in which individual development accounts with matched savings and financial literacy trainings were added to HIV risk reduction (intervention arm 1); and a second intervention arm in which a vocational skills training and mentorship intervention were added to the first study arm (intervention arm 2). However, because of a countrywide lockdown as a result of the unfolding COVID-19 pandemic, implementation of the vocational skills training and mentorship intervention in the second intervention arm was disrupted. Subsequently, the study protocol was amended, and the two treatment arms were merged into one. The trial is registered under the clinical trials registry (ClinicalTrials.gov registration no. NCT02406482), and the study protocol detailing the study design and procedure is published elsewhere.^29^ The study protocol was approved by the Uganda Virus Research Institute Ethics Committee (GC/127/18/10/690), the Uganda National Council for Science and Technology (SS4828), the Washington University in St. Louis Institutional Review Board (#201811106), and the Columbia University Institutional Review Board (AAAR9804).

A total of 542 women were enrolled in the study from 19 hot spots in Masaka, Kalungu, Kyotera, Rakai, and Mpigi districts, located in the Greater Masaka and Rakai regions in southern Uganda. The main economic activities in the districts include crop agriculture, animal ranching, and fishing. Communities living in these districts are fraught with socioeconomic disparities, and there are a notable number of orphaned children and women living in poverty, a legacy of the civil wars in the 1970s and 1980s.^31^ Masaka is also the district where the first HIV patient was detected in Uganda in 1982.^32^ Despite the national HIV prevalence among 15- to 49-year-olds being 7.2%, Rakai (9.3%) and Masaka (12%) districts report higher HIV prevalence than the national average.^29^ Particularly, the HIV prevalence among WESW in Rakai and Masaka districts is estimated to be as high as 61%.^33^ Antiretroviral therapy has been provided free of charge in Uganda since 2004.^34^ Although the national threshold for good adherence to ART is 84% and above, a recent study in the study districts estimated self-reported ART adherence at 72.8% among WESW.^35^

Participation in the study was voluntary, and written consent from participants was obtained before they participated in the study. The Kyaterekera study began in September 2018 and planned to continue for a period of 64 months, until December 2023, to collect follow-up data on the participants. For the purposes of this costing analysis, we used the resource use and cost data collected during the delivery of the intervention between September 2018 and December 2022. The age range of participants at enrollment was 18–54 years, and all participants reported having engaged in vaginal or anal intercourse in the past 30 days in exchange for money or other goods and to have at least one unprotected intercourse during the same period.^36^ A cluster randomization approach using each hot spot as a cluster allocated the participants to one of the two study arms. In brief, the 19 hot spots were initially randomly assigned to three study arms. Later, owing to the COVID-19 pandemic, two of these arms were merged to become the intervention arm, whereas one remained as the control arm. Further details on the study design can be found in the study protocol paper.^29^ As a result, the control arm included 186 women (7 clusters) who received a traditional HIV risk-reduction intervention, and the intervention arm included 356 women (12 clusters) who received a matched savings account and financial literacy trainings as an economic empowerment intervention on top of the traditional HIV risk-reduction intervention.

### Control arm.

Participants in the control arm received four sessions of an evidence-based HIV risk-reduction intervention from trained community health workers (CHWs) over the course of 2 weeks. The effectiveness of HIV risk-reduction interventions in reducing HIV/STI risks has been proven in previous studies conducted in similar settings.^16^^,^^19^^,^^20^ Guided by the social cognitive theory,^37^ the HIV risk-reduction intervention provides education on HIV/STI risk-reduction strategies, including but not limited to condom use, safe communication and refusal, and recognizing partners’ abusive behaviors. During these sessions, skills training to improve ART medication adherence was also provided by CHWs while linkage to pre-exposure prophylaxis was provided by medical personnel who joined these sessions, facilitated by CHWs. In addition, educational materials on HIV/AIDS, developed and provided by the Ugandan Ministry of Health, were distributed to all participants.

### Intervention arm.

Participants in the intervention arm first received four sessions of HIV risk-reduction training over the course of 2 weeks as described above. At the beginning of the intervention, all participants in this arm also received an individual development account, which was a matched savings account at a local bank with an initial deposit. Throughout the intervention period, deposits made by participants were matched one-to-one to incentivize saving behavior and asset building. After the HIV risk-reduction training, participants also received six training sessions on financial literacy from program staff over a 6-week period. Financial literacy training followed an adapted format of an evidence-based financial education core curriculum^38^ and behavioral economics principles,^39^ previously administered in our studies targeting young women in Uganda,^18^^,^^27^ and provided education on banking, financial goal setting, budgeting, debt management, emergency financing, and safe sexual and income-earning practices.

### Costs data collection and analysis.

We used a prospective micro-costing approach to evaluate the economic costs of the interventions under each study arm. This approach enumerates all resources consumed by program activities for the delivery of interventions and is considered a more accurate method of resource-use assessment in economic analyses of interventions compared with macro-costing approaches, which identify costs at an aggregated level.^40^ Hence, resource use in each study arm was prospectively collected throughout the study period to ensure the reliability and validity of the cost estimates. Unlike financial costs, economic costs include the value of leveraged resources, including donated goods and volunteered hours of work, and hence are more informative for policymakers and program officers.^41^ This costing analysis considers and values all leveraged resources and adopts the program provider perspective so that the results focus on economic costs relevant to the implementation of interventions in real-life settings. By doing so, we further enhanced the utility of the study findings to inform planning and budgeting for implementation and prioritization and resource allocation in the context of alternative interventions and limited public health resources.

To identify all program-related activities involved in each study arm, we reviewed the study protocol and the administrative documents of the program. We also conducted periodic interviews with key program staff. Program-related activities included identification of study hot spots and study sites, screening and recruitment of study participants, training of HIV risk-reduction facilitators, delivery of HIV risk-reduction sessions by trained CHWs, delivery of financial literacy training sessions by program staff, opening of individual development accounts, and stakeholder engagement and dissemination. We excluded the costs of any research-specific activities from the analysis, including baseline and follow-up assessments of participants that aim to assess the effectiveness of the Kyaterekera intervention. By doing so, we limited the scope of our analysis to the resources used in program delivery in a nonresearch setting, making our findings more useful for policymakers and program officers to assess and estimate resource use requirements and costs in their own implementation settings.

We then prospectively measured and valued all resources used for each activity or cost category (Table 1). Shared resources and costs across the two study arms included personnel, program overhead (utilities, transportation, communication, maintenance, and office supplies), donated resources (time spent on community mobilization for recruitment of study participants) and stakeholder engagement and dissemination meetings, and capital costs (furniture, equipment, vehicles); they were measured and valued as separate cost categories and apportioned to the study arms. Time costs of program staff were calculated based on average annual gross wage rates and number of hours each staff member devoted to all activities each year. Relative use of staff time for program (versus research) activities in each study arm was determined based on periodic interviews with key program staff, and the estimated time costs for research activities were excluded from the analysis. Other recurring costs included time compensation of CHWs for delivery of HIV risk-reduction sessions, participation incentives for study participants, facilitation incentives provided to site coordinators for mobilization of study participants, venue costs for HIV risk-reduction and financial literacy training sessions, time donated to program activities by site coordinators, time donated for individual development account opening by bank officials, initial deposits into individual development accounts, and provision of matched savings. Participation incentives were provided to study participants to cover their travel and any other participation costs so as to encourage and maintain their participation in the program in this low-income setting. We incorporated the costs of donated time for program activities to arrive at the economic costs of the intervention, capturing the opportunity costs associated with time spent by site coordinators and bank officials for the implementation of program activities, representing the foregone net benefit of allocating that time to alternative purposes. All pertinent expenditures and the time spent by site coordinators and bank officials on program-related activities were extracted from the program’s financial and administrative records. Similarly, to capture capital expenditures made for the program, capital costs were differentiated from recurrent costs and were valued and annualized using the purchasing price and replacement cost of each capital item, an appropriate useful life for each item, and a recommended annual discount rate.^42^

**Table 1:** Identification, quantification, and valuation of main cost categories

Cost Category	Cost Items	Measurement and Calculation Methods
Personnel	On the basis of administrative and financial expenditure records of the study, the time devoted to program activities (versus research activities) in each study arm by program staff	Extract the number of hours each staff member devoted to all activities each year, multiply the total hours by average hourly salary rate of staff (estimated based on average annual gross wage rate), and calculate the total cost across all staff. Apportion the total cost incurred each year based on level of effort dedicated by staff to program activities (80%) (versus research, 20%) and to each study arm (10% control and 90% intervention), and divide it by the number of participants in each study arm.
Identification of study sites and site visits	Facilitation incentives for site coordinators	Divide total cost incurred each year by the number of participants in each study arm.
Screening and recruitment of study participants	Facilitation incentives for site coordinators to mobilize study participants, participation incentives for study participants, interviewer compensations for screening of participants	Divide total cost incurred each year by the number of participants in each study arm.
Usual care: delivery of HIV risk-reduction sessions	Participation incentives for participants, time compensation for CHWs for HIV risk-reduction session implementation, facilitation incentives for site coordinators, venue used for HIV risk-reduction sessions	Divide total cost of delivering HIV risk-reduction sessions each year by the number of participants in each study arm.
Training of HIV risk-reduction facilitators	Time compensation for CHWs	Divide total cost incurred by the number of participants in each study arm.
Individual development accounts	Account opening, initial deposit, matched contributions	Divide total cost of opening individual development accounts and the total amount of matched contributions by the number of participants in the intervention arm.
Delivery of financial literacy training sessions	Participation incentives for participants, facilitation incentives for site coordinators, venue used for financial literacy training sessions	Divide total cost of delivering the workshop each year by the number of participants in the intervention arm.
Donated resources	Time spent by site coordinators on mobilization of participants for program-related activities, time spent by bank officials for individual development account opening	Based on an hourly time cost of each site coordinator and bank officials, calculate the total cost incurred each year. Apportion the total cost to each study arm, and divide it by the number of participants in each study arm.
Program overheads	Utilities (water, electricity, communication), transportation (fuel, taxi fare, car hire), security services and insurance, maintenance (equipment, vehicles, and facilities), materials and office supplies, and other miscellaneous costs	Apportion the total cost incurred each year based on the level of effort dedicated by staff to program activities (80%) (versus research, 20%) and to each study arm (10% control and 90% intervention), and divide it by the number of participants in each study arm.
Stakeholder engagement and dissemination	Induction seminars and stakeholder meetings (transport refund for attendees, venue rental, materials, and office supplies)	Divide total cost incurred from each year by the number of participants in each study arm.
Capital costs	Capital items with an expected useful life of more than 1 year, such as equipment, vehicles, and furniture	Calculate equivalent annual costs by annualizing all capital costs over the useful life of capital items (3 years for equipment, 5 years for furniture, and 10 years for vehicles) using an annual discount rate of 3%, and apportion the total cost incurred from each year based on the level of effort dedicated by staff to program activities (80%) (versus research, 20%) and to each study arm (10% control and 90% intervention), and divide it by the number of participants in each study arm.

CHW = community health worker.

Once all resources were appropriately valued, we calculated the total cost per participant per study arm. To do so, first, all costs were adjusted for inflation using the Ugandan Consumer Price Index,^43^ discounted at an annual rate of 3%^44^ to the start year of the study and presented in 2019 US dollars. The costs of shared resources were apportioned to program- and research-related activities using a ratio of 80:20. This ratio was conservatively derived based on interviews with key program staff. To apportion the costs of the shared resources to each study arm, we derived an apportioning ratio by dividing the costs of intervention activities in each arm by the total costs of all intervention activities across the two arms. The arm-specific costs were then summed and added to the costs of the apportioned shared resources to derive the total cost per study arm. To arrive at the per-participant cost per study arm, the total cost per study arm was then divided by the number of participants in the respective arm using the intent-to-treat (ITT) and per-protocol samples. Although the ITT sample comprised all randomized participants according to randomized treatment assignment and ignored anything that happened after randomization, including noncompliance, protocol deviations, and withdrawal, the per-protocol sample was based on the number of participants who actually participated in the study and may therefore be smaller than the ITT sample. Therefore, cost calculations using the per-protocol sample typically yielded higher and more conservative per-participant cost estimates. A conservative approach to costing is always preferable in economic analyses of interventions as it errs on the side of caution by providing a margin of error around cost estimates and yields more conservative cost-effectiveness estimates. A summary of identified program activities, cost items, and measurement and calculation methods is presented in Table 1.

## RESULTS

**Figure 1.**
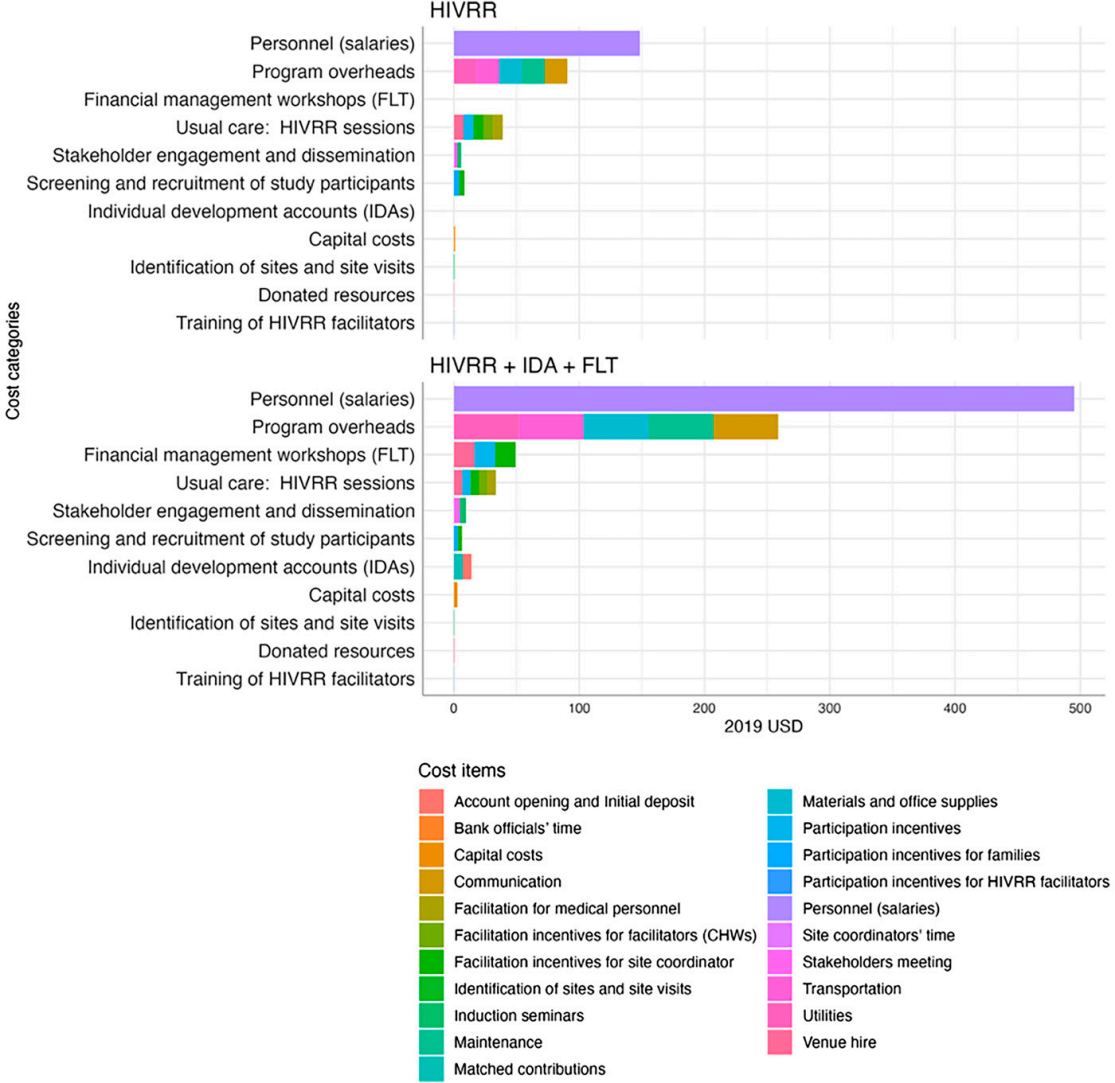
Cost per participant breakdown by study arm using the intent-to-treat (ITT) sample. CHW = community health worker; FLT = financial literacy training; HIVRR = HIV risk-reduction intervention.

Tables 2 and 3 and Figure 1 present the per-participant costs by cost category and by study arm (control arm, *N* = 186, and intervention arm, *N* = 356) using the ITT sample. The per-participant costs for the study arms were estimated at $183 for the control arm and at $588 for the intervention arm. The incremental cost of the intervention arm over the control arm was $405 per participant. For both study arms, personnel costs accounted for the largest proportion of the total costs, followed by program overheads. In the intervention arm, the costs of financial management workshops and individual development accounts were the underlying cost drivers, following the costs of personnel and program overheads. For the control arm, the cost of HIV risk-reduction sessions drove the costs, following the costs of personnel and program overheads.

**Table 2:** Per-participant costs by study arm using the intent-to-treat sample

Costs	Control Arm	Intervention Arm
Personnel (salaries)	531,825	1,774,392
Identification of study sites and site visits	1,203	807
Facilitation incentives for site coordinators	1,203	807
Screening and recruitment of study participants	15,035	11,725
Participation incentives for study participants	13,402	10,575
Facilitation incentives for site coordinators	1,632	1,150
Usual care: HIV risk-reduction sessions	27,933	24,033
Participation incentives for study participants	14,815	13,191
Facilitation incentives for HIV risk-reduction facilitators	7,651	6,977
Facilitation incentives for site coordinators	2,444	891
Facilitation for medical personnel	989	722
Venue rental	2,034	2,252
Individual development accounts	–	25,082
Account opening and initial deposits	–	8,315
Matched contributions	–	16,766
Financial literacy training sessions	–	58,902
Participation incentives for study participants	–	54,310
Facilitation incentives for site coordinators	–	1,958
Venue rental	–	2,634
Training of HIV risk-reduction facilitators	567	542
Participation incentives for HIV risk-reduction facilitators	567	542
Donated resources	441	850
Site coordinators’ time	441	561
Bank officials’ time	–	289
Program overheads	64,885	185,469
Utilities	4,685	11,954
Transportation	47,410	138,744
Communication	2,638	5,105
Maintenance	7,113	22,475
Materials and office supplies	3,039	7,190
Stakeholder engagement and dissemination	10,183	17,129
Stakeholder meetings	3,569	13,823
Induction seminars	6,614	3,307
Capital costs	3,599	10,264
Total cost	655,671	2,109,197
Total cost (in 2019 US dollars)	183	588

All costs are in 2019 Ugandan shillings unless otherwise indicated.

**Table 3:** Total and per-participant costs by study arm using the intent-to-treat sample

	Control Arm (*n* = 330)		Intervention Arm (*n* = 660)	
	Total Cost	Total Cost per Participant	Total Cost	Total Cost per Participant
Personnel (salaries)	48,926.64 (81.1%)	148.26	326,479.58 (84.1%)	494.67
Identification of sites and site visits	110.71 (0.2%)	0.34	148.55 (0.04%)	0.23
Screening and recruitment of study participants	1,383.17 (2.3%)	4.19	2,157.36 (0.6%)	3.27
Usual care: HIV risk-reduction sessions	2,569.73 (4.3%)	7.79	4,422.00 (1.1%)	6.70
Individual development accounts	–	–	4,614.95 (1.2%)	6.99
Financial management workshops	–	–	10,837.77 (2.8%)	16.42
Training of HIV risk-reduction facilitators	52.14 (0.1%)	0.16	99.80 (0.03%)	0.15
Donated resources	40.55 (0.1%)	0.12	156.47 (0.04%)	0.24
Program overheads	5,969.31 (9.9%)	18.09	34,125.37 (8.8%)	51.71
Stakeholder engagement and dissemination	936.81 (1.6%)	2.84	3,151.74 (0.8%)	4.78
Capital costs	331.11 (0.5%)	1.00	1,888.56 (0.5%)	2.86
Total	60,320.18	182.79	388,082.16	588.00

All costs are in 2019 US dollars.

**Figure 2.**
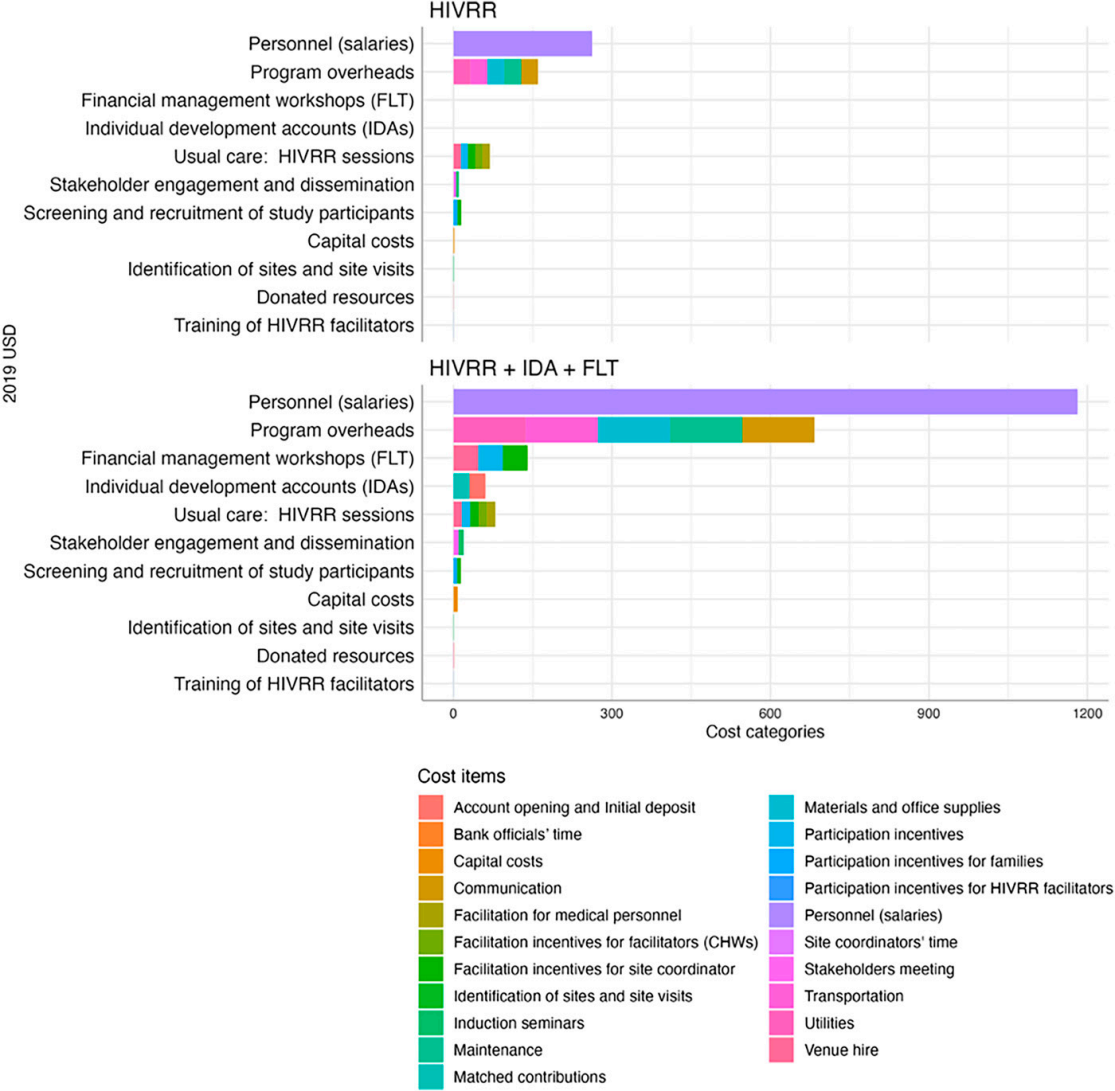
Cost per participant breakdown by study arm using the per-protocol sample. CHW = community health worker; FLT = financial literacy training; HIVRR = HIV risk-reduction intervention.

**Figure 3.**
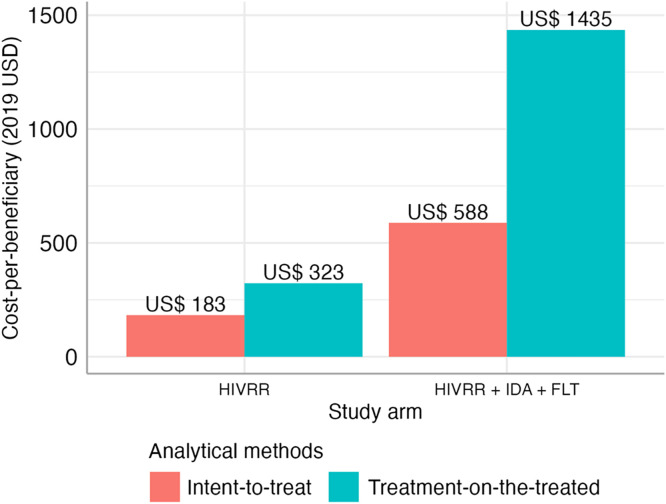
Per-participant costs by study arm per analytical method. FLT = financial literacy training; HIVRR = HIV risk-reduction intervention; IDA = individual development account.

Tables 4 and 5 and Figure 2 present the per-participant costs by cost category and study arm using the per-protocol sample, which are based on the actual number of WESW who participated in each study arm and hence are more conservative cost estimates. The per-participant cost estimates based on the per-protocol sample were therefore higher across the two study arms compared with those estimated using the ITT sample and were $323 and $1,435 for the control and intervention arms, respectively. The incremental cost of the intervention arm over the control arm was $1,112 per participant. Figure 3 compares the per-participant costs per study arm by analytical method.

**Table 4:** Per-participant costs by study arm using the per-protocol sample

Costs	Control Arm	Intervention Arm
Personnel (salaries)	941,065	4,233,807
Identification of study sites and site visits	2,135	1,497
Facilitation incentives for site coordinators	2,135	1,497
Screening and recruitment of study participants	26,614	25,008
Participation incentives for study participants	23,725	22,567
Facilitation incentives for site coordinators	2,889	2,440
Usual care: HIV risk-reduction sessions	49,415	56,703
Participation incentives for study participants	26,227	29,459
Facilitation incentives for HIV risk-reduction facilitators	13,523	16,552
Facilitation incentives for site coordinators	4,320	2,355
Facilitation for medical personnel	1,750	1,750
Venue rental	3,595	6,587
Individual development accounts	–	108,250
Account opening and initial deposits	–	23,916
Matched contributions	–	84,334
Financial literacy training sessions	–	167,467
Participation incentives for study participants	–	152,938
Facilitation incentives for site coordinators	–	6,179
Venue rental	–	8,350
Training of HIV risk-reduction facilitators	1,006	1,006
Participation incentives for HIV risk-reduction facilitators	1,006	1,006
Donated resources	780	2,145
Site coordinators’ time	780	1,301
Bank officials’ time	–	844
Program overheads	114,672	489,832
Utilities	8,273	37,218
Transportation	83,805	350,964
Communication	4,650	20,922
Maintenance	12,581	56,602
Materials and office supplies	5,363	24,127
Stakeholder engagement and dissemination	18,059	34,588
Stakeholder meetings	6,325	28,457
Induction seminars	11,734	6,131
Capital costs	6,360	28,614
Total cost	1,160,106	5,148,916
Total cost (in 2019 US dollars)	323	1,435

All costs are in 2019 Ugandan shillings unless otherwise indicated.

**Table 5:** Total and per-participant costs by study arm using the per-protocol sample

Costs	Control Arm (*n* = 188)		Intervention Arm (*n* = 356)	
	Total Cost	Total Cost per Participant	Total Cost	Total Cost per Participant
Personnel (salaries)	48,797.24 (81.1%)	262.35	420,187.98 (82.2%)	1,180.30
Identification of sites and site visits	110.71 (0.2%)	0.60	148.55 (0.03%)	0.42
Screening and recruitment of study participants	1,380.02 (2.3%)	7.42	2,481.91 (0.5%)	6.97
Usual care: HIV risk-reduction sessions	2,562.34 (4.3%)	13.78	5,627.54 (1.1%)	15.81
Individual development accounts	–	–	10,743.35 (2.1%)	30.18
Financial management workshops	–	–	16,620.38 (3.3%)	46.69
Training of HIV risk-reduction facilitators	52.14 (0.1%)	0.28	99.80 (0.02%)	0.28
Donated resources	40.45 (0.1%)	0.22	212.88 (0.04%)	0.60
Program overheads	5,946.09 (9.9%)	31.97	48,613.80 (9.5%)	136.56
Stakeholder engagement and dissemination	936.43 (1.6%)	5.03	3,432.70 (0.7%)	9.64
Capital costs	329.80 (0.5%)	1.77	2,839.86 (0.6%)	7.98
Total	60,155.21	323.42	511,008.75	1,435.42

All costs are in 2019 US dollars.

## DISCUSSION

To achieve the United Nation’s target of eliminating HIV/AIDS by 2030, prioritization of effective interventions to reduce HIV risk among WESW is essential. However, challenges in implementing and sustaining programs for WESW, attributed to stigma, their high mobility, and a heightened level of mistrust,^45^^,^^46^ limit the evidence base on the feasibility, affordability, and sustainability of interventions and programs for this key population. This is the first study to investigate the costs of a combination intervention that added a proven HIV risk-reduction intervention to an evidence-based savings-led economic empowerment intervention to simultaneously address economic hardship and HIV risk among WESW in Uganda.

We showed that at an incremental cost of US $400–US $1,200 per WESW, traditional HIV risk-reduction approaches can be augmented by an economic empowerment intervention. As a result, the estimated cost of the combination intervention ranged between US $550 and US $1,450 per WESW (Figure 3). Although the upper limit of the cost range is much higher than the costs of other combination interventions aimed at reducing HIV risk in different target populations, the estimated range is still aligned with the published cost range of $504 (in 2015 US dollars) to $890 (in 2016 US dollars) per beneficiary for such interventions.^17^^,^^47^^–^^49^ The major drivers of the intervention costs were the cost of opening individual development accounts and the matched deposits, followed by personnel and transportation costs for program activities. The latter was anticipated given WESW’s higher mobility and geographically dispersed hot spots.^29^^,^^50^ In addition, difficulty in sustaining WESW in the program after initial recruitment resulted in a substantial reduction in participation and hence the size of the per-protocol sample, further increasing the upper limit of the cost range. The unique factors associated with the higher cost of the intervention are worth noting for program implementers and policymakers who plan to prioritize and implement such interventions for this key population. Further, per-participant cost estimates were derived based on 4 years of program operation, which included setup costs in the first year. This suggests that when planning for program implementation on an annual basis, the program budget should include a higher first year budget to consider and incorporate the setup costs of the program.

Given the dearth of evidence, this study lays a solid foundation for future studies that aim to assess the economic costs of HIV combination interventions targeting WESW in low-resource settings. Utilizing an activity-based micro-costing approach and standardized costing tools,^51^ the study captured actual resource use, providing policymakers and implementers with reliable and context-specific cost estimates of the intervention.^52^

This study has a number of limitations. First, instead of real-time monitoring of time spent on all activities through daily logs or observations, we opted for periodic interviews with key program staff throughout the study. Although this method reduced the burden on program staff and minimized interference with program implementation, we acknowledge that it is an important study limitation, affecting the reliability of our cost estimates. In future studies, program staff and others who contribute time to intervention activities could complete daily logs, which would contribute to a more accurate understanding of how they allocate time across intervention and research activities.

Second, this study was conducted as part of an evaluation study alongside a large-scale longitudinal clinical trial. As such, although we excluded research-related activities, such as baseline and follow-up assessments of study participants, the shared nature of some resources (e.g., personnel, program overheads, donated resources, capital items) was unavoidable. Based on the information provided by multiple program staff and our understanding of our unique implementation and research context and associated activities, we used a conservative ratio of 80% (versus research, 20%) to apportion the shared resources to program activities so as to estimate the resource requirements of the intervention in a nonresearch setting. However, despite our best efforts, we cannot rule out that some costs could have been under- or overestimated under this umbrella approach.

Third, although our choice of the provider perspective ensured the relevance of the captured resource requirements and costs for this new intervention’s replication and quality implementation in real-life settings, we recognize the value of adopting a societal perspective in costing and cost-effectiveness analyses. This broader perspective includes all costs regardless of who pays for them, including nonhealthcare costs incurred by households, such as nonmedical costs (e.g., meals, transportation, lodging), labor productivity losses (i.e., wages forgone), and informal caregiver costs, as well as costs beyond the health sector.^44^ This becomes especially pertinent when considering the potential use of societal or public funds for program purposes, a scenario that is likely if this intervention were to be scaled up. On the other hand, although comprehensive, the societal perspective aggregates costs across all perspectives and limits understanding of the economic implications of implementation specific to each stakeholder group.^53^ Therefore, future studies should consider, collect, and report resource requirements and costs from multiple perspectives to support the adoption, implementation, and sustainment of evidence-based interventions, particularly in low-resource settings.

Lastly, it is important to restate that each implementation context is unique. Our study estimated the economic costs of a combination intervention that integrated an HIV risk-reduction intervention with an economic empowerment intervention that specifically targeted WESW living in five districts in southern Uganda. Although the unique socioeconomic context of our geographical setting and the characteristics of WESW are extensively described in our previous publications,^4^^,^^54^^,^^55^ our findings may not be generalizable across different geographic and implementation settings and program delivery modalities. It is for this reason that we used a prospective activity-based micro-costing method to map the resources to specific intervention activities, which allows for the application of our costing framework and findings to other similar implementation settings.

## CONCLUSION

Thorough cost analysis is inescapably complex, yet such analysis sets the stage for further assessment of the feasibility, affordability, and sustainability of public health interventions and programs, particularly in low-resource communities. Particularly, accurate costing is crucial for economic evaluation of interventions and programs to guide prioritization and resource allocation decisions and to advocate for further investment to bring them to scale in areas where the need is greatest. Lastly, although the per-beneficiary cost of the Kyaterekera intervention was estimated to be higher than that of similar combination interventions targeting other key populations in Uganda and other low-income settings, it is crucial to recognize that achieving health equity is equally important. Simply relying on cost considerations is likely to perpetuate longstanding disparities, leaving behind marginalized populations, including WESW.^56^^,^^57^ However, it is important that decision-making integrates evidence on both costs and effectiveness to inform prioritization of public health interventions. In this light, although we argue that our findings should not discourage the consideration of combination interventions targeting WESW simply because of their higher implementation costs, their prioritization would ultimately depend on their effectiveness and cost-effectiveness in resource-constrained settings. To that end, our study contributes to the dearth of evidence on the economic costs of combination interventions to facilitate their economic evaluation and highlights the importance of investments in marginalized populations to address HIV-related disparities and health inequities through proven interventions and programs.

## Data Availability

We confirm that the data supporting the findings of this study are available within the article. Raw data were generated at the International Center for Child Health and Development (ICHAD) and are available from the corresponding author on request.
